# Reducing the Impact of Shoulder Abduction Loading on the Classification of Hand Opening and Grasping in Individuals with Poststroke Flexion Synergy

**DOI:** 10.3389/fbioe.2017.00039

**Published:** 2017-06-30

**Authors:** Yiyun Lan, Jun Yao, Julius P. A. Dewald

**Affiliations:** ^1^Interdepartmental Neuroscience Program, Northwestern University, Chicago, IL, United States; ^2^Department of Physical Therapy and Human Movement Sciences, Northwestern University, Chicago, IL, United States; ^3^Department of Biomedical Engineering, Northwestern University, Chicago, IL, United States; ^4^Department of Physical Medicine and Rehabilitation, Northwestern University, Chicago, IL, United States

**Keywords:** stroke, flexion synergy, machine learning, hand movements, classification, neural machine interface, coherence

## Abstract

Application of neural machine interface in individuals with chronic hemiparetic stroke is regarded as a great challenge, especially for classification of the hand opening and grasping during a functional upper extremity movement such as reach-to-grasp. The overall accuracy of classifying hand movements, while actively lifting the paretic arm, is subject to a significant reduction compared to the accuracy when the arm is fully supported. Such a reduction is believed to be due to the expression of flexion synergy, which couples shoulder abduction (SABD) with elbow/wrist and finger flexion, and is common in up to 60% of the stroke population. Little research has been done to develop methods to reduce the impact of flexion synergy on the classification of hand opening and grasping. In this study, we proposed a novel approach to classify hand opening and grasping in the context of the flexion synergy using a wavelet coherence-based filter. We first identified the frequency ranges where the coherence between the SABD muscle and wrist/finger flexion muscles is significant in each participant, and then removed the synergy-induced electromyogram (EMG) component with a subject-specific and muscle-specific coherence-based filter. The new approach was tested in 21 stroke individuals with moderate to severe motor impairments. Employing the filter, 14 participants gained improvement in classification accuracy with a range of 0.1 to 14%, while four showed 0.3 to 1.2% reduction. The remaining three participants were excluded from comparison due to the lack of significant coherence, thus no filters were applied. The improvement in classification accuracy is significant (*p* = 0.017) when the SABD loading equals 50% of the maximal torque. Our findings suggest that the coherence-based filters can reduce the impact of flexion synergy by removing the synergy-induced EMG component and have the potential to improve the overall classification accuracy of hand movements in individuals with poststroke flexion synergy.

## Introduction

Functional movements that demand independent joint control of the shoulder, elbow, and wrist/fingers (e.g., reach-to-grasp) are essential to activities of daily living. Unfortunately, most individuals with chronic hemiparetic stroke have lost such ability due to the stereotypical muscle coactivation patterns between shoulder abductor, elbow flexor, and wrist/finger flexors, commonly referred to as the flexion synergy (Dewald et al., 1995; Sukal et al., 2007; Miller and Dewald, 2012; Lan et al., 2014; Ellis et al., 2016). Due to the expression of the flexion synergy, many individuals find it harder or even impossible to open the hand and/or grasp an object when lifting the paretic arm at the same time (Miller and Dewald, 2012; Lan et al., 2014). To overcome this difficulty, past studies have implemented statistical models to learn and translate the electrical biosignals [e.g., electroencephalogram (EEG) or electromyogram (EMG)] into control signals of external devices, such as robotic exoskeletons (Collinger et al., 2013; Hortal et al., 2015) or functional electrical stimulation systems (Moritz et al., 2008; Pohlmeyer et al., 2009; Ethier et al., 2012). While moderate to high accuracies in learning and translating the poststroke EMGs were reported (Sang et al., 2010; Zhang and Zhou, 2012), none of them have given consideration of the deleterious effect of the flexion synergy.

Due to the flexion synergy, poststroke EMG signals recorded at wrist and fingers during functional movements include two components (Miller and Dewald, 2012; Lan et al., 2016). One component is the voluntary EMG signals generated due to the voluntary contraction of wrist and finger muscles; and the other one is the synergy-induced EMG signals generated due to the involuntary contraction of wrist and fingers muscles associated with the activation of shoulder abductor muscles (Miller and Dewald, 2012; Lan et al., 2014). It has been shown that after a stroke synergy-induced EMG signals from the impaired hand can reach to a significant level with increased shoulder abduction (SABD) loading, even when a study participant was instructed to relax the hand (Miller and Dewald, 2012). These synergy-induced EMGs do not represent the intention of hand movements and thus are detrimental to the accurate classification of volitional hand movements (Lan et al., 2011). For example, when the impaired arm was fully supported, i.e., no effect of flexion synergy, the classification of hand movements can reach an overall accuracy of 96% with high-density myoelectric recordings (Zhang and Zhou, 2012), or 95% with bipolar surface EMG recordings (Lan et al., 2011), but when lifting the paretic arm, the overall accuracy drops by 10 to 16% using EMG signals (Lan et al., 2011) or using EEG signals (Yao et al., 2015).

The overall goal of this study is to find out whether it is possible to reduce the impact of the flexion synergy on the classification accuracy of hand movements by removing the synergy-reduced EMG signals from the wrist and fingers muscles. It was noted from earlier findings that voluntary EMGs and synergy-induced EMGs may be generated using different neural pathways. The synergy-induced EMG signals are likely to be delivered *via* slow-conducting, polysynaptic contralesional corticoreticulospinal pathway, resulting in EMG–EMG oscillation in the alpha band (8–13 Hz) between muscles that share the same neural projections (Lan et al., 2016). In contrast, voluntary EMGs are conveyed *via* fast-conducting, monosynaptic corticospinal pathway that produces EMG–EMG oscillation in the beta band (15–30 Hz) (Farmer et al., 1993; Gross et al., 2000; Lan et al., 2016). With coherence analysis of EMGs between the shoulder abductor and wrist/finger muscles, it is possible to differentiate the synergy-induced EMGs from the voluntary EMGs by studying the coherence power during hand movements. It is our hypothesis that the overall classification accuracy in individuals with stroke should be improved after removing the synergy-induced EMGs with a specific filter. Such a filter should be coherence-based and subject-specific due to the expected between-subject variation in the frequency ranges where the coherence of synergy-induced EMGs is significant. Classification accuracies before and after the filtering will be compared and discussed.

## Materials and Methods

### Participants

A total of 29 individuals (stroke: 21, control: 8) participated in this study. Participant demographics are listed in Table 1. Control participants were age-matched to the stroke participants and reported no history of cerebral vascular accidents. Stroke participants were selected from the Clinical Neuroscience Research Registry that is housed in the Rehabilitation Institute of Chicago, as well as from individuals residing in the Chicago area who wished to participate in the study. Qualified stroke participants met the following inclusion criteria: (1) sustained a unilateral lesion at least 1 year prior to participation in this project; (2) paresis confined to one side; (3) absence of a brainstem and/or cerebellar lesion; (4) absence of severe concurrent medical problems (e.g., cardiorespiratory impairment, changes in management of hypertension); (5) absence of any acute or chronic painful condition in the upper extremities or spine; (6) capacity to provide informed consent; (7) ability to elevate their limb against gravity up to horizontal and to generate some active elbow extension; and (8) Fugl-Meyer Assessment (Fugl-Meyer et al., 1975) within the range of 10–40 out of a possible 66 and 2–5 out of a possible 7 in Chedoke-McMaster Stroke Hand Assessment (Gowland et al., 1993). All subjects gave informed consent for participation in this study, which was approved by the Institutional Review Board of Northwestern University in accordance with the ethical standards stipulated by the 1964 Declaration of Helsinki for research involving human subjects.

**Table 1:** Participant demographics.

	Stroke	Control
Age (years)	59 ± 9 (40–71)	55 ± 12 (42–83)
Gender (M/F)	15/6	5/3
Time since stroke (years)	11 ± 7 (1–28)	
Sides of tested UE^a^ (L/R)	17/4	0/8
UE FMA	26 ± 10 (12–39)	
CMSAh	3 ± 1 (2–5)	

*M, male; F, female; L, left; R, right; UE, upper extremity; FMA, Fugl-Meyer assessment; CMSAh, Chedoke-McMaster Stroke Assessment (hand)*.

*Values are listed as mean ± SD (range)*.

*^a^In this experiment, the stroke subjects were tested at the paretic UE, while the control subjects were tested at the dominant UE*.

### Equipment and Setup

**Figure 1:**
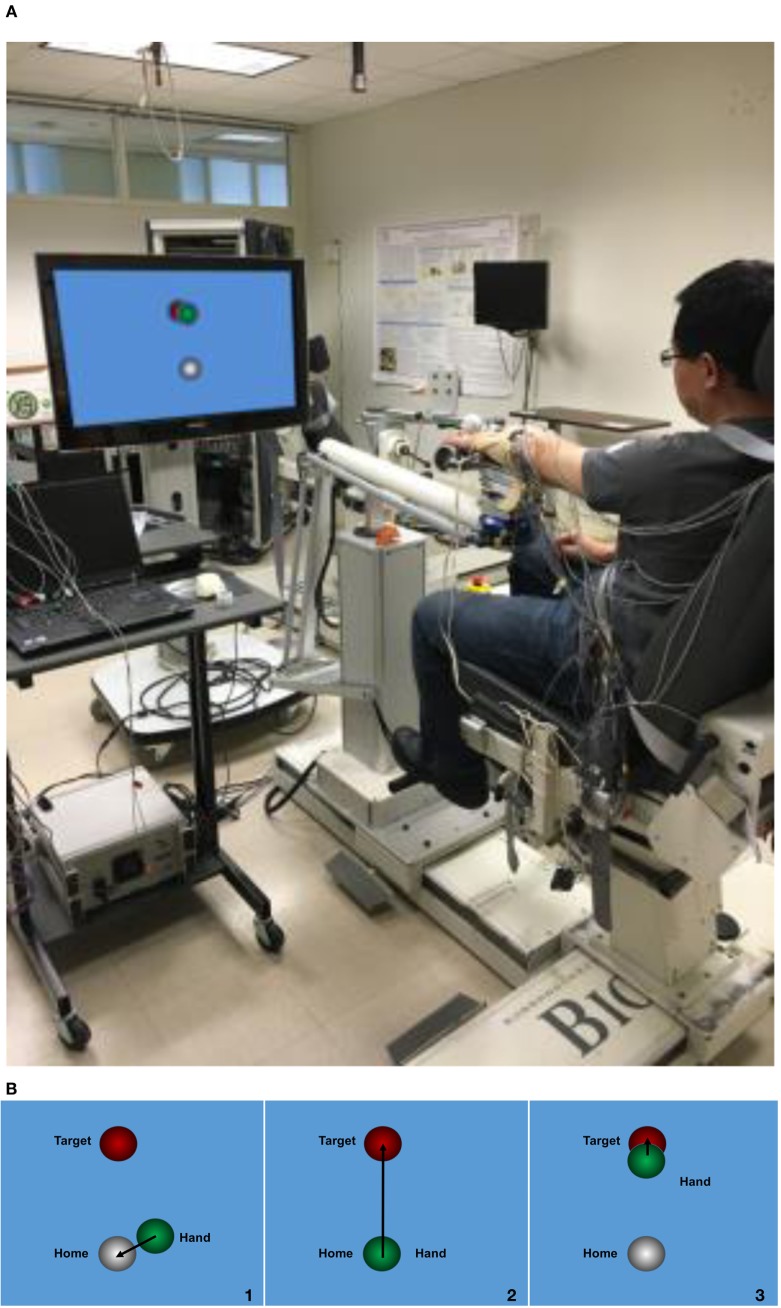
Experiment setup. **(A)** ACT^3D^ system with a monitor display; **(B)** visual feedback during the task, step 1: to find the home position; step 2: found the home position and triggered the trial; step 3: to find the target position.

The experiment was carried out using an arm coordination training 3-D system (ACT^3D^, Figure 1A), which consists of a modified HapticMaster robot (Moog-FCR B. V., the Netherlands), a Biodex chair (Biodex Medical Systems, Shirley, NY, USA), and T-base support system (Biodex Medical Systems, Shirley, NY, USA). The ACT^3D^ allows for free movements in three dimensions and was used to modulate SABD torques applied to the tested arm.

For the experimental setup, each participant was seated in the Biodex chair with the trunk strapped to the back of the chair to prevent unwanted movement of the upper body. The to-be-tested forearm was placed in a forearm orthosis and the fingers/palm rested on a cylinder. The cylinder was rigidly coupled to the end effector of the ACD^3D^. Surface EMGs were collected using an Avatar physiological recorder (Electrical Geodesics, Inc., Eugene, OR, USA) from intermediate deltoid (mDEL), flexor carpi radialis (FCR), flexor digitorum superficialis (FDS), extensor carpi radialis (ECR), and extensor digitorum communis (EDC). EMGs were sampled at 1,000 Hz and preprocessed with a band-pass filter at a cutoff band of 5–450 Hz.

### Protocol

Prior to the experiment, each participant’s maximum SABD torque was measured using a manual dynamometer (Lafayette Instrument Company, Lafayette, IN, USA) placed just proximal to the axis of rotation of the elbow in a limb configuration of 85° SABD, 45° shoulder flexion, and 90° elbow flexion. Participants were presented with a home object and a target object on a monitor in front of them (Figure 1B). At the beginning of the task, participants were instructed to find the home object, trigger the trial, and then reach out to the target object. Once the hand arrived at the target, the participant was given 2 s to lift the tested arm and hold the position. After the 2 s, while keeping the arm lifted, the participant was asked to perform one of the following three hand tasks for 5 s in a random order: (1) open the hand with a maximal effort; (2) grasp the cylinder with a maximal effort; (3) no hand movement. All participants performed these three hand tasks with two different SABD loadings equaling to 25 or 50% of the subject’s maximum SABD torque. Ten to twelve repetitions of each hand task were performed.

### Data Analysis

#### Coherence

Wavelet coherence was used to examine the linear dependency of two sequences of surface EMGs in the time-frequency domain (Torrence and Compo, 1998) and is considered efficient and reliable in detecting the synchronizing activity between two time series (Daubechies, 1990; Jevrejeva et al., 2003; Grinsted et al., 2004). In this study, the Morlet wavelet was applied for transformation. Monte Carlo simulation methods were used to determine the 5% statistical significance level of the coherence (Grinsted et al., 2004). It is assumed that the EMG time series has a mean power spectrum, which is only considered as significant when it is above the white noise of the background spectrum. And during the holding phase of grasping/opening, the coherence remains relatively steady (Baker et al., 1997; Kilner et al., 1999, 2000). Wavelet coherences were calculated for each of following muscle pairs, i.e., FDS-mDEL, FCR-mDEL, EDC-mDEL, and ECR-mDEL, for each participant and for all three hand tasks.

#### Algorithm

##### Without Coherence-Based Filter

All EMG signals were manually segmented and concatenated to exclude the idling EMGs collected between hand tasks. A 250-ms long window was implemented to slide from the beginning to the end of the concatenated EMGs with a 50% increment and a 50% overlap between adjacent moving windows. Within each moving window, features were extracted based on the method proposed by Hudgins (Hudgins et al., 1993). This method proposes four features in the time domain: mean absolute value, zero crossings, slope sign changes, and waveform length (see Table 2 for the definition of each of these four features).

**Table 2:** Electromyogram features extracted in the time domain.

Features	Description
Zero crossing	*x_k_ x_k_*_+1_ < 0 and |*x_k_* − *x_k_*_+1_| ≥ ∈
Slope sign changes	{*x_k_* > *x_k_*_−1_ and *x_k_* > *x_k_*_+1_} or {*x_k_* < *x_k_*_−1_ and *x_k_* < *x_k_*_+1_} and |*x_k_* − *x_k_*_+1_| ≥ ∈ or |*x_k_* − *x_k_*_−1_| ≤ ∈
Absolute amplitude	$x_{i}=\frac{1}{L}\overset{L}{\underset{k=1}{\sum }}\left|x_{k}\right|\text{for }i=1,2,\ldots ,L$
Waveform length	$l_{0}=\overset{L}{\underset{k=1}{\sum }}\left|\Delta x_{k}\right|$

*k, the kth sample; x, the feature; ∈, pre-defined threshold; L, window length; |Δx_k_|, waveform length between two adjacent samples*.

Linear discriminant analysis (LDA) was used to classify the EMG signals in this study. LDA has been proved to be effective in EMG classification as well as with low computational cost (Scheme and Englehart, 2011). For each of the hand tasks, LDA maximizes the posterior probability of Bayesian equation and assigns the class labels (i.e., hand open, grasp, or relax) with the largest possibility,

$$\underset{j}{\text{argmax}}p(y_{j}|x_{ij})=\frac{p\left(x_{ij}\text{|}y_{j}\right)\text{*}p\left(y_{j}\right)}{\sum_{i=1,j=1}^{i=n,j=m}p\left(x_{ij}\text{|}y_{j}\right)\text{*}p\left(y_{j}\right)}$$

where *x_ij_* and *n* represent the features and the number of features in the training set, *i* indicated the *i*th feature, *j* indicated the *j*th category, *y* is classification category.

##### With Coherence-Based Filter

Frequency ranges where coherence was significant were first identified during the three hand tasks. For each participant, significant ranges found in the three hand tasks were merged to determine the cutoff frequency for the band-stop filters (fourth order Butterworth). Separate filters were built specific to each of the two SABD loading levels in this study. Concatenated EMG signals were then preprocessed with these coherence-based filters followed by feature extraction and LDA classification described above.

#### Dataset and Model Evaluation

For each participant, the dataset was split into a training set (75%) and a testing set (25%). The training set was used to train the model to learn and differentiate the EMG patterns of different hand tasks. The model was trained and tested based on a 250-ms long window. Each subject will generate 10–12 trials of 5 s hand movements for three hand tasks (open/grasping/relax). The total number of the dataset is 1,200–1,440 samples. Tenfold cross-validation was implemented to determine the best model that reported the highest detection accuracy in training set. The testing set was then used to assess the strength of the model and the extent to which the fitted model could generalize to the future data. The testing set was put aside until the model was complete with training.

Model was evaluated by calculating the classification accuracy within each of hand movement categories, as shown below,

$$\text{accuracy}=\frac{\text{number of correct classification}}{\text{total number of classification}}\%.$$

The overall classification accuracy is the mean classification accuracy of the three hand tasks.

#### Statistics

A mixed three-way ANOVA with repeated measures was conducted to determine whether loading (SABD25, SABD50), filter (before filtering, after filtering), hand task (relax, open, grasp), and/or their interaction explain the changes in classification accuracies. *Post hoc* comparisons with the Bonferroni adjustment were used to compare within-group differences. Unless specified otherwise, results are presented as mean ± SE. Statistical significance was set at *p* < 0.05. The statistical analysis was performed using the IBM SPSS version 22 software.

## Results

**Figure 2:**
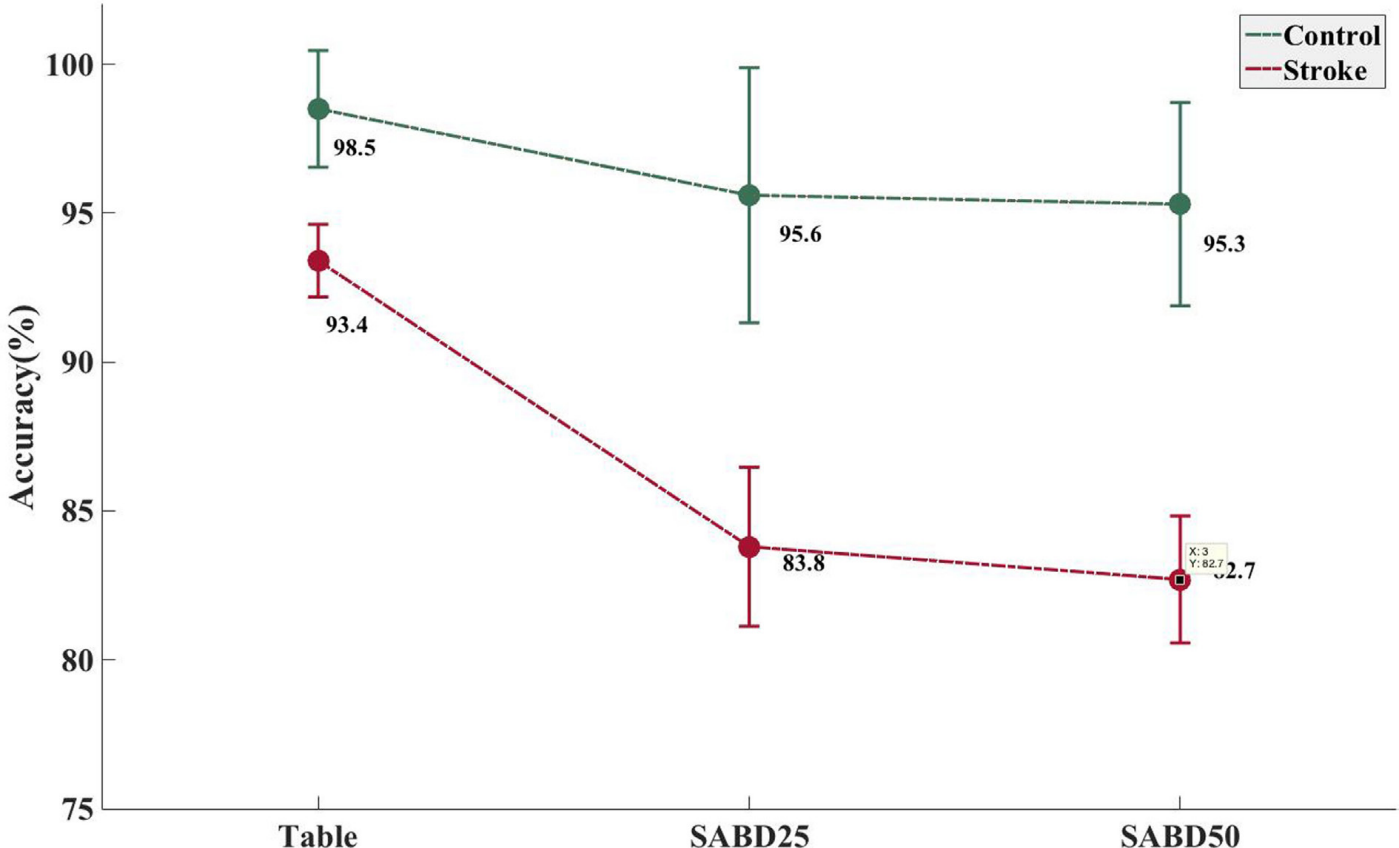
Increased shoulder abduction (SABD) loading resulted in a significantly decreased accuracy rate in the stroke group. Mean and SE of classification error rate in the stroke group (*N* = 21) and in the able-bodied group (*N* = 8). Table, participant’s tested arm was fully supported on a rigid flat surface; SABD25 and SABD50, participant lifted the tested arm with a weight that equaled to 25 and 50% of his/her maximal SABD torque, respectively.

Shoulder abduction loading showed a negative impact on the overall accuracy in the stroke group (Figure 2, see *p* values in Table 3). Both groups showed high overall accuracy when the tested arm was fully supported, but the accuracy in the stroke group started to drop significantly during conditions where the participants lifted the paretic arm thus generating SABD loads. The control group was not significantly affected by SABD loading.

**Table 3:** Mixed two-way ANOVA for overall accuracy rate.

Main effect and interaction	
Factor	Overall accuracy rate
Group	*p* = 0.009
Loading	*p* = 0.000
Loading × group	*p* = 0.048

***Post hoc* analysis**	

**Loading**	**Group (control vs stroke)**

TABLE	*p* = 0.093
SABD25	*p* = 0.015
SABD50	*p* = 0.011

**Group**	**Loading (TABLE, SABD25, SABD50)**

Stroke	*P*_tb-25_ = 0.000
	*P*_25–50_ = 1.000
	*P*_tb-50_ = 0.000
Control	*P*_tb-25_ = 1.000
	*P*_25–50_ = 0.245
	*P*_tb-50_ = 1.000

**Figure 3:**
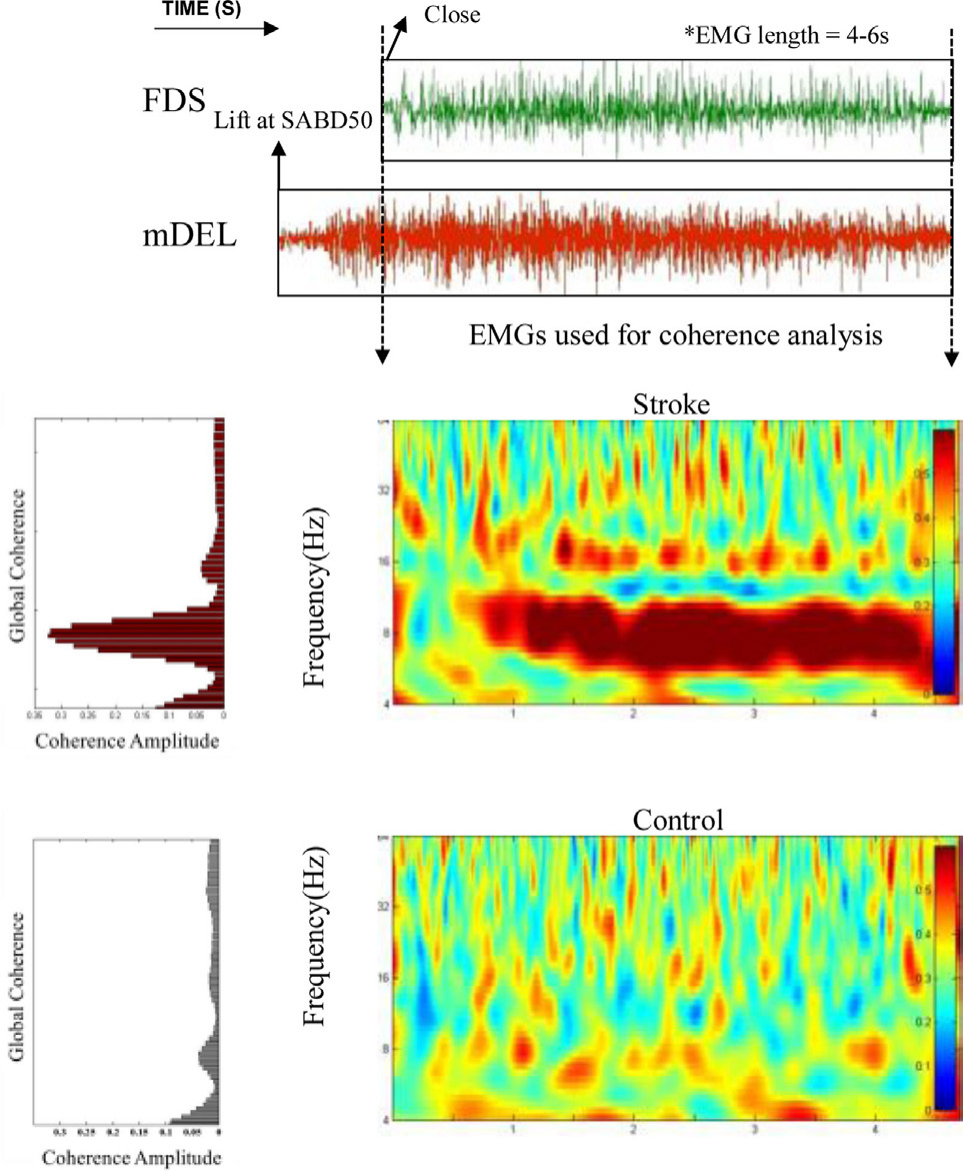
Greater alpha-band coherence between mDEL and wrist/finger flexors in the stroke individual. Top: poststroke electromyogram (EMG) signals from flexor digitorum superficialis (FDS) and mDEL were presented during the hand grasp task while the stroke participant was lifting the paretic arm at the same time (SABD50); Middle: wavelet coherence was calculated with the aligned EMG signals for the stroke participant, and the global coherence was plotted on the left side to show the coherence power aggregating over time; Bottom: wavelet coherence and global coherence for a control individual (EMG signals for this control individual are not shown).

The result of coherence analysis of EMGs is shown for one stroke participant and one able-bodied individual during grasping while generating a 50% of max SABD load (see Figure 3). Significant coherence between wrist/finger flexors and mDEL in the alpha band (8–13 Hz) was found in the stroke participant during the hand grasp task while such activities were mostly absent in the able-bodied individual (Figure 3). The increased alpha-band coherence during hand grasping while lifting the arm in the stroke individual suggested a greater level of shared neural drive to both muscles. The global coherence depicted the coherence power aggregated over time as a function of frequency, highlighting the frequency range where coherence power was the most prominent. It is evident from the global coherence figures that this stroke individual had a greater level of EMG–EMG synchronization that centered at around 8 Hz.

Our data also show a significant global coherence between wrist/finger muscles and mDEL for the hand grasping task with SABD loading at 50% of the max torque based on the Monte Carlo simulation approach in each of the participants (see Figure S1 in Supplementary Material). For each participant, the significant frequency range is represented by solid lines whose lengths denote the range and a solid filled circle whose location denotes the peak value. The figure shows that the stroke group has more significant coherence bands in the alpha band than the control group, especially for the more severely impaired individuals. Coherence in the beta band is not evident for the stroke group. Additionally, there is also great variation between subjects and between muscles in the alpha band coherence in the stroke group. For example, the significant coherence in the extensors are either very short (ECR for the severe cases) or very rare (EDC for the severe cases), while coherence in the flexors is generally longer. Across all individuals, no one stroke individual shared the same significant frequency band as the other.

**Figure 4:**
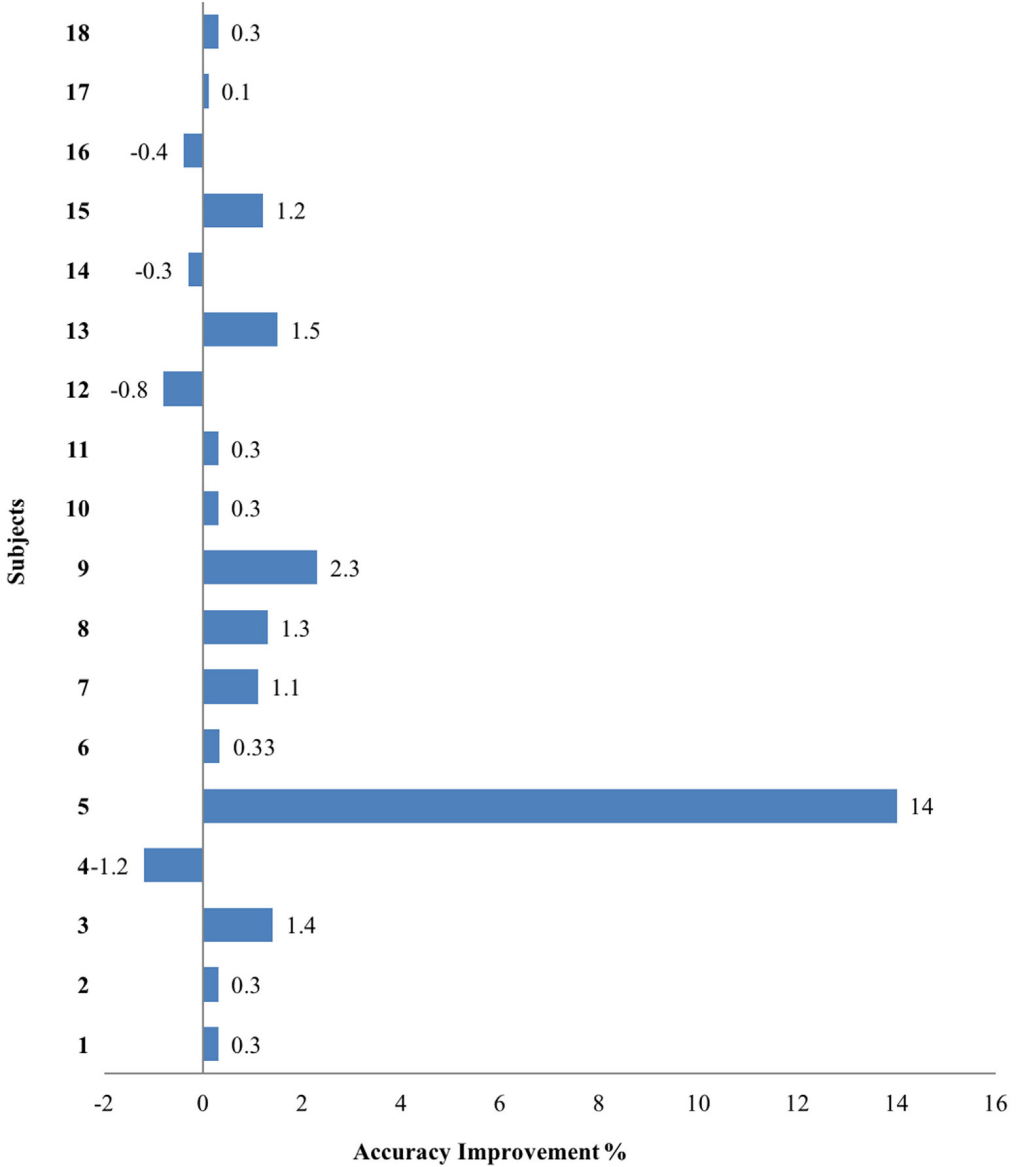
The overall classification accuracy improvement after applying the filters at SABD50 in the stroke group. Positive and negative values indicate improvement and reduction in the accuracy after filtering, respectively.

Based on the significant coherence bands found in all three hand tasks, the cutoff frequency for the band-stop filter was determined by the frequency ranges where coherence was significant for each muscle in each participant. This subject-specific, muscle-specific coherence-based filter (referred as “coherence filter” below) was applied to the EMGs in each participant to remove the synergy-induced components from the original EMGs. For individuals (*n* = 3) who showed the peak value as the only significant coherence or no significant bands, no coherence filter was applied. Results from these three individuals were not included in the statistic analysis either. Figure 4 showed the improvement in classification accuracy after applying the coherence filters to the stroke group at SABD50. Overall, 14 subjects showed improvement in the classification accuracy, four subjects showed reduced accuracy after removing the synergy-induced EMGs. A mixed three-way ANOVA found significance in an interaction effect of loading × filters (*p* < 0.05). *Post hoc* analysis found no significant difference in the classification accuracy with the coherence filters applied at SABD25, but significantly greater improvement with the coherence filters at SABD50 (Table 4). One subject showed 14% improvement with the filter. It is expected that some individuals may respond very favorably to the filter. Nevertheless, even when eliminating this individual from the analysis, the conclusion still holds as the sample mean at SABD50 without this subject remains significant compared to the one without the filter (*p* = 0.038).

**Table 4:** Mixed three-way ANOVA for improvement in the overall accuracy rate.

Main effect and interaction	
Factor	Improvement
Filter	*p* = 0.05
Task × filter	*p* = 0.106
Loading × filter	*p* = 0.048
Task × loading × filter	*p* = 0.160

***Post hoc* analysis**	

**Loading**	**Group (control vs stroke)**

SABD25	*P*_with_filter − no_filter_ = 0.443
SABD50	*P*_with_filter − no_filter_ = 0.017

## Discussion

### Novelty and Main Finding

Decoding EMG signals for neural machine interface is a great challenge in the individuals with chronic hemiparetic stroke due to prevalent motor deficits, such as flexion synergy (Lan et al., 2011; Yao et al., 2015). Many studies made great efforts acquiring better quality of EMGs or features to improve the overall classification accuracy. For example, Zhang and Zhou (2012) have reported that using high-density EMG signals can achieve high classification accuracies in the stroke individuals. Englehart and Hudgins (2003) suggested optimal parameters for feature extraction, such as window length, overlap, increment length to produce models with low bias, and variances that can generalize well to the test data (Scheme and Englehart, 2011). Features in the time domain (Hudgins et al., 1993; Zhou et al., 2007), the frequency domain (Merletti, 1997; Li et al., 2014), and the time-frequency domain (Englehart et al., 1999; Zhou et al., 2007; Nurhazimah et al., 2016) have also been broadly investigated. While improvement of the overall accuracy has been reported, the limitation is that very few studies have given full consideration of the effect of the flexion synergy common in up to 60% of the stroke population nor proposed approaches that can reduce EMG contamination associated with activation of proximal arm muscles (Fougner et al., 2011; Lan et al., 2011).

This study proposed a novel approach to reduce the impact of the flexion synergy on classification of the hand movements in individuals with chronic hemiparetic stroke for future use in neural machine interfaces. A subject-specific and muscle-specific coherence-based filter was developed to remove the synergy-induced component in EMG signals collected from the forearm. The subject-specific filter is believed to be more effective in removing individualized synergy-induced EMG component than a filter with a universal cutoff frequency as the expression of flexion synergy on the frequency ranges of significant coherence varies across individuals with different motor impairment severities (see Figure S1 in Supplementary Material). It was found that such filters can significantly improve the classification accuracy during a greater level SABD loading. To our knowledge, this is the first study in the field that took the effect of the upper-limb flexion synergy during functional reaching, hand opening, and grasping tasks into consideration.

### Impact of Flexion Synergy on Classification Accuracy

It was previously reported that SABD loading had a negative impact on the overall classification accuracy of hand opening using either EMGs (Lan et al., 2011) or EEGs (Yao et al., 2015). Even in the able-bodied individuals, variations in the limb position can have a substantial impact on the robustness of EMG recognition (Fougner et al., 2011). The results in this study confirm these previous findings. It is now understood that the activation of shoulder abductors can result in the involuntary coactivation of the wrist and fingers (Miller and Dewald, 2012), and such involuntary expression of flexion synergy at the hand can be further enhanced by increased SABD loading on the paretic limb. The EMG signals in the flexors increased with SABD loading even when no voluntary hand movement was initiated (Miller and Dewald, 2012). Prior studies suggested that the synergy-induced EMG component might be delivered *via* the contralesional corticoreticulospinal pathway during increasing levels of SABD (Dewald et al., 1995; Miller and Dewald, 2012; Lan et al., 2016) and should not represent the volitional aspect of hand movement, thus resulting in a decrease in classification accuracy. It is worth noting that while the paretic limb was fully supported, the overall classification of the stroke group has an average of 93.4% accuracy, suggesting that the current feature extraction and classifier choice is sufficient to decode myoelectric patterns in the absence of flexion synergy. However, the same feature extraction and classifier choice is less effective in the presence of synergy-induced EMG, such as when lifting the weight of the arm. It also seems that the reduction in classification accuracy, due to increased SABD loading, is not strictly linear since the accuracy at SABD50 only decreased by 1.1% compared with SABD25. However, more and greater SABD loading conditions are needed to confirm the relationship between SABD loading and reduction in classification accuracy.

### Variation in Significant Coherence Frequency Range

For both hand grasp and hand open tasks, there was great between-subject and between-muscle variation in the frequency ranges where the coherence between wrist/finger muscles and mDEL is significant. One explanation is the broad range of stroke severity included in this study. The severity of stroke participants in this study ranges from moderate to severe impairment, as demonstrated by Fugl-Meyer Assessment and Chedoke-McMaster Stroke Hand Assessment (see methods). It is possible that more severe individuals show greater coherence in the alpha band due to the increased reliance on the contralesional corticoreticulospinal pathway, resulting in a greater portion of synergy-induced EMGs at the wrist/fingers during SABD. The between-muscle variation is also evident across individuals. For example, the significant coherence frequency range in the flexors is generally more common than the extensors (Figure S1 in Supplementary Material). It was noted from previous studies that hand muscles receiving projections from the contralesional reticulospinal tract are flexor-facilitated on the impaired side (Davidson and Buford, 2006; Riddle et al., 2009), meaning activation of the flexors using this pathway is much stronger than the extensors. From evidence provided in monkeys which had recovered from a unilateral lesion of the pyramidal tract, it was shown that reticulospinal-induced amplitude and incidence of synaptic inputs to forearm flexors were significantly increased, while inputs to extensors remained unchanged (Baker et al., 2015). For the control group, the significant coherence frequency range is no more than sporadic across all muscles and subjects, indicating the reduced extent of shared neural drive to the shoulder and the hand compared to stroke participants.

### Variation in Classification Improvement

Electromyogram classification from some individuals (e.g., stroke participant 9, see Figure S1 in Supplementary Material) responded more favorably to the filtering process than others (e.g., stroke participant 1). Such difference may be related to the remained volume of ipsilesional corticospinal tract that is responsible for voluntary hand movements. It is possible that individuals with a more intact ipsilesional corticospinal tract may have smaller room of improvement in classification accuracy. Conversely, individuals with great reliance on the contralesional corticoreticulospinal tract may benefit more from the coherence filter after the synergy-induced EMGs was removed. It is also important to point out that three stroke individuals showed zero improvement. This is because none of them showed significant coherence frequency ranges and, therefore, no filters were applied. Interestingly, four individuals showed reduced classification accuracy after applying the filters. This could be due to the artifact introduced by the filters that may have caused EMG signal attenuation, and EMGs from these four individuals may be particularly sensitive to such an artifact. It could also be due to the fact that these four individuals already had limited voluntary EMGs in the first place thus removing the synergy-induced component brought little change in the overall classification accuracy.

### Scientific Implications and Future Work

A common approach to preparing surface EMGs is to apply a band-pass filter with a cutoff frequency range of 20–450 Hz. This is very much rooted in the previous work by De Luca and colleagues demonstrating that most of the energy related to motion artifacts is in the frequency range from 0 to 20 Hz (De Luca, 2002; De Luca et al., 2010). However, more recent evidence has shown that after stroke the central nervous system might have adopted an alternative motor control strategy that generates neural oscillation in the alpha range (Lan et al., 2014; Baker et al., 2015). This control strategy may emphasize using neural pathways that produce frequency contents under 20 Hz and hence it was suggested as the potential target for poststroke rehabilitation (Baker et al., 2015). That could imply that the EMG below 20 Hz might contain useful motor information. Thus *selectively* removing the frequency content below 20 Hz might be a more effective approach for pattern recognition of poststroke EMGs and should be studied in more detail. To achieve this goal, future work should first focus on quantifying real-time classification in the stroke group. The present work is an off-line application of the algorithm, and we reported the overall improvement in most stroke individuals. To make this approach more clinically applicable, it is recommended to implement the algorithm online, meaning classification is made while EMGs are generated. We have used a 250ms-long time window for processing the data. This allows for an online classification of the hand movement without creating a sense of delay. Second, the classification platform should be realized with a close-loop connection to an external device (e.g., a robot device or functional electrical stimulator), which receives the classification signal and generates movements or activates relevant muscles in the wrist and fingers. Eventually, such a platform requires a training period which acquires subject-specific data to train the classifier and a real-time testing period.

Future work should also extend the current experimental protocol to multiple abduction levels to better understand the benefits of applying filters to functional hand movements. Furthermore, integrating neuroimaging approaches into the current line of research can further help us to gain scientific insight into the possible use of ipsilesional corticospinal and contralesional corticoreticulospinal tracts after stroke. This is likely to bring new perspectives to a more effective subject-specific future application of neural machine interfaces within the context of flexion synergy.

## Ethics Statement

This study was carried out in accordance with the recommendations of the Institutional Review Board of Northwestern University with written informed consent from all subjects. All subjects gave written informed consent in accordance with the Declaration of Helsinki. The protocol was approved by the “the Institutional Review Board of Northwestern University.”

## Author Contributions

YL designed the study, developed the methodology, performed the analysis, and wrote the manuscript under the guidance of JY and JD. Both JY and JD contributed extensively to revising and finalizing this manuscript as well as to the development of the original research idea.

## Conflict of Interest Statement

The authors declared no potential conflicts of interest with respect to the research, authorship, and/or publication of this article.
