# Supplementary material for: Reducing the Impact of Shoulder Abduction Loading on the Classification of Hand Opening and Grasping in Individuals with Poststroke Flexion Synergy
Source: Front Bioeng Biotechnol. 2017 Jun 30;5:39. doi: 10.3389/fbioe.2017.00039 (PMC5491847; doi:10.3389/fbioe.2017.00039)
Supplement: Supplementary file 1 [file Image_1.PDF]

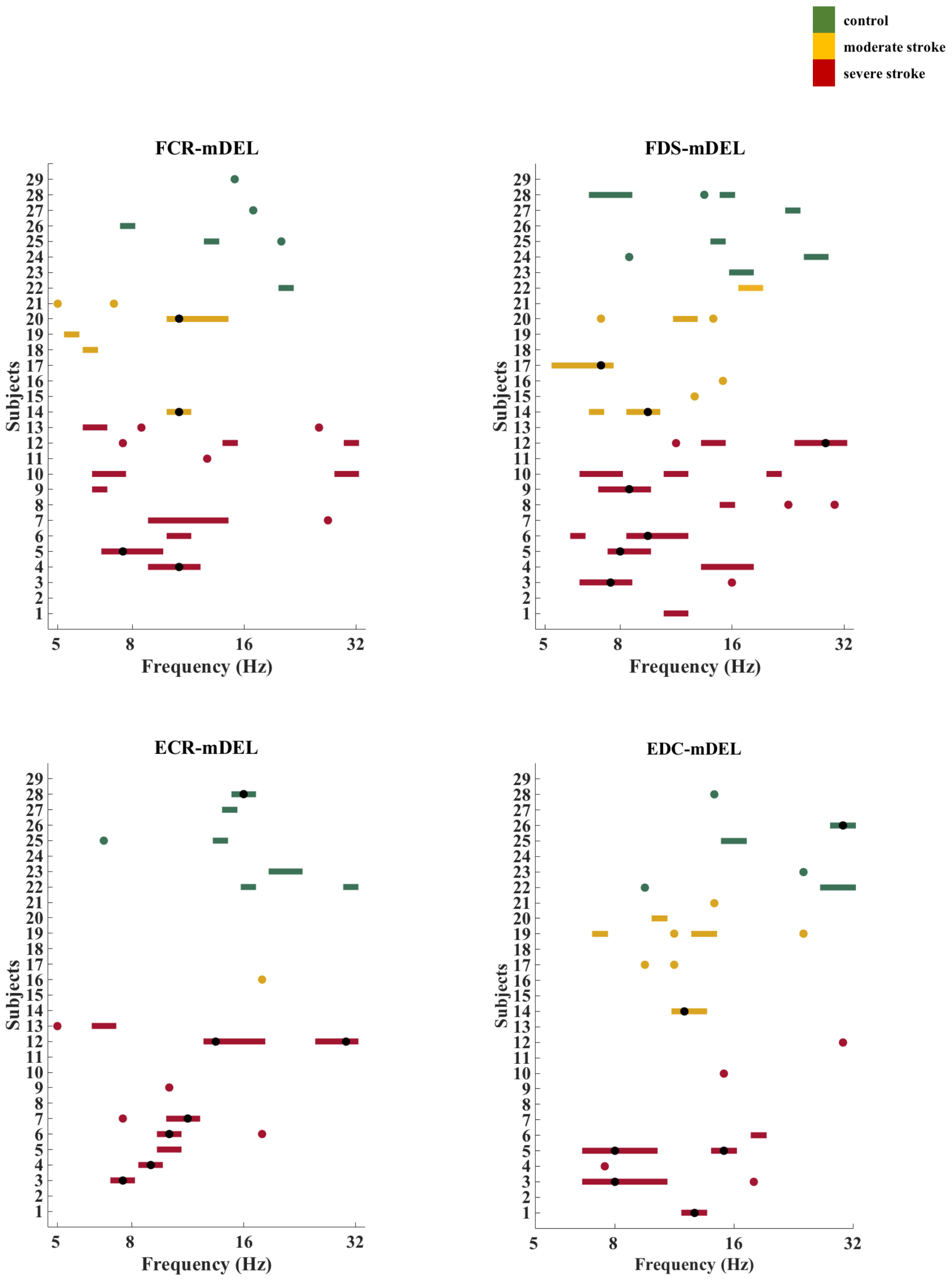

Complementary Figure. Significant frequency range across participants during hand grasping at SABD50.
